# Pattern of HIV risk behavior in a cohort of high risk women in East Africa

**DOI:** 10.1186/1742-4690-9-S2-P124

**Published:** 2012-09-13

**Authors:** HN Kibuuka, K Rono, L Maganga, M Millard, A Sekiziyivu, L Maboko, D Shaffer, A Valenzuela, N Michael, M Robb

**Affiliations:** 1 Makerere University-Walter Reed Project, Kampala, Uganda; 2 Walter Reed Project, Kericho, Kenya; 3 Mbeya Medical Research Program, Mbeya, Tanzania, United Republic of; 4 US Military HIV Research Program, Bethesda, MD, USA

## Background

Development of high risk cohorts is critical for advanced HIV vaccine testing. Prevention interventions during follow up of such cohorts could influence the pattern of risk behavior leading to unmet study outcomes. We examined risk behaviors among high risk women enrolled in a prospective cohort to determine changes over time.

## Methods

Adult women self identified as sex workers or bar workers were enrolled in an open cohort at three sites in East Africa. HIV risk factors were assessed at baseline and every six months for 1½ years, using Audio Computer Assisted Self Interview (ACASI). Participants were also evaluated twice weekly to identify HIV infection. HIV counseling was done every 3 months and when required during twice weekly visits. Male condoms were made available at all visits. Data was analyzed using Fisher’s exact test.

## Results

Data is available for 1158 HIV negative participants at baseline and 771(66.6%), 537 (46.4%), 403 (34.8 %) participants at 6, 12 and18 months respectively and 37 acute HIV infections. Overall, the risk status of Tanzania women was lower compared to Kenya and Uganda. There was a significant drop in proportion of participants reporting sex with ≥ 3 Non-spouse/Non-cohabitating male partners and sex with high risk partners at 6 months (25.3%, 39.0%) compared to baseline (55.4 %, 62.0%)( p< 0.0001, <0.0001 ) but no decline subsequently. Most participants (76.8%) used alcohol during sex with male partners at baseline and throughout the study (73.3%, 72.8%, and 72.9% at 6, 12 and 18 months). There was a significant increase in proportion of participants using condoms at 6 months in Tanzania (p<0.006). Incidence rates were 3.3, 2.7 and 1.5 per 100PYs during 0-6, >6-12 and >12-18 months.

## Conclusion

HIV prevention interventions among high risk individuals may result in significant decreases in risky behavior that could have implications for future trials.

